# Dashboards in Health Care Settings: Protocol for a Scoping Review

**DOI:** 10.2196/34894

**Published:** 2022-03-02

**Authors:** Danielle Helminski, Jacob E Kurlander, Anjana Deep Renji, Jeremy B Sussman, Paul N Pfeiffer, Marisa L Conte, Oliver J Gadabu, Alex N Kokaly, Rebecca Goldberg, Allison Ranusch, Laura J Damschroder, Zach Landis-Lewis

**Affiliations:** 1 Department of Internal Medicine University of Michigan Ann Arbor, MI United States; 2 Institute for Healthcare Policy and Innovation University of Michigan Ann Arbor, MI United States; 3 Veterans Affairs Ann Arbor Center for Clinical Management Research Ann Arbor, MI United States; 4 Department of Learning Health Sciences University of Michigan Ann Arbor, MI United States; 5 Department of Psychiatry University of Michigan Ann Arbor, MI United States; 6 Taubman Health Sciences Library University of Michigan Ann Arbor, MI United States; 7 Department of Medicine UCLA Health Los Angeles, CA United States; 8 University of Michigan Medical School Ann Arbor, MI United States

**Keywords:** dashboard, mHealth, medical informatics, quality improvement, scoping review, health care, Cochrane library, Cochrane, stakeholder, health care sector, digital health, design, end user, development, implementation, evaluation, user need

## Abstract

**Background:**

Health care organizations increasingly depend on business intelligence tools, including “dashboards,” to capture, analyze, and present data on performance metrics. Ideally, dashboards allow users to quickly visualize actionable data to inform and optimize clinical and organizational performance. In reality, dashboards are typically embedded in complex health care organizations with massive data streams and end users with distinct needs. Thus, designing effective dashboards is a challenging task and theoretical underpinnings of health care dashboards are poorly characterized; even the concept of the dashboard remains ill-defined. Researchers, informaticists, clinical managers, and health care administrators will benefit from a clearer understanding of how dashboards have been developed, implemented, and evaluated, and how the design, end user, and context influence their uptake and effectiveness.

**Objective:**

This scoping review first aims to survey the vast published literature of “dashboards” to describe where, why, and for whom they are used in health care settings, as well as how they are developed, implemented, and evaluated. Further, we will examine how dashboard design and content is informed by intended purpose and end users.

**Methods:**

In July 2020, we searched MEDLINE, Embase, Web of Science, and the Cochrane Library for peer-reviewed literature using a targeted strategy developed with a research librarian and retrieved 5188 results. Following deduplication, 3306 studies were screened in duplicate for title and abstract. Any abstracts mentioning a health care dashboard were retrieved in full text and are undergoing duplicate review for eligibility. Articles will be included for data extraction and analysis if they describe the development, implementation, or evaluation of a dashboard that was successfully used in routine workflow. Articles will be excluded if they were published before 2015, the full text is unavailable, they are in a non-English language, or they describe dashboards used for public health tracking, in settings where direct patient care is not provided, or in undergraduate medical education. Any discrepancies in eligibility determination will be adjudicated by a third reviewer. We chose to focus on articles published after 2015 and those that describe dashboards that were successfully used in routine practice to identify the most recent and relevant literature to support future dashboard development in the rapidly evolving field of health care informatics.

**Results:**

All articles have undergone dual review for title and abstract, with a total of 2019 articles mentioning use of a health care dashboard retrieved in full text for further review. We are currently reviewing all full-text articles in duplicate. We aim to publish findings by mid-2022. Findings will be reported following guidance from the PRISMA-ScR (Preferred Reporting Items for Systematic Reviews and Meta-Analyses extension for Scoping Reviews) checklist.

**Conclusions:**

This scoping review will provide stakeholders with an overview of existing dashboard tools, highlighting the ways in which dashboards have been developed, implemented, and evaluated in different settings and for different end user groups, and identify potential research gaps. Findings will guide efforts to design and use dashboards in the health care sector more effectively.

**International Registered Report Identifier (IRRID):**

DERR1-10.2196/34894

## Introduction

### Background

Effectively measuring, monitoring, and responding to metrics about health-related decisions, practices, and outcomes has become an essential business function for modern health care organizations. Nimble health care organizations employ data for all manners of daily operational decision-making, ranging from supply chain management and staff scheduling to individual treatment planning and population health management [1]. For certain key performance metrics, payers have linked reimbursement to value-based payment programs [2] and accrediting bodies have required monitoring and disclosure of performance for accreditation or certification [3], incentivizing organizations to effectively monitor and track their performance against established benchmarks [4]. With the rapid proliferation of electronic health records, there is an abundance of patient- and provider-level data to use for assessing performance [5-7]. At the same time, vast data alone are of little use without systems to derive timely and actionable insights.

Health systems have increasingly adopted business intelligence software to track performance metrics in an automated way [8]. These applications have been defined by Loewen and Roudsari [9] as “specialized tools to collect, analyze, and present organizational data to operational leaders in user-friendly format(s) to support organizational objectives.” One such tool that has seen considerable expansion in health care settings is the “dashboard,” a business intelligence tool that uses data visualization to provide actionable feedback to improve performance, adherence to evidence-based practices, workflow management, and resource utilization [10,11]. Dashboards often display performance trends, peer comparisons, benchmarks, or goals, and use visual elements such as graphs and color-coding to improve interpretability [12].

To create an effective dashboard, developers must make multiple complex decisions. End users’ information needs are highly contextual and depend on the clinical setting, professional roles, and the patient population, which impact selection of appropriate data elements, visualizations, and interactivity [13-15]. Although health care executives may prefer to see graphic performance trends over weeks or months, clinicians working with vulnerable patient groups may require real-time, patient-level health data so they can intervene quickly if needed. Indeed, numerous techniques for developing dashboards and selecting key metrics have been described, including focus groups, iterative usability testing, and a Delphi method [16,17]. More sophisticated dashboards also incorporate forecasting and decision support, which carry their own challenges [18,19]. The range of and most common strategies used to address these essential steps in dashboard development are unknown.

Developing effective dashboards tailored to the needs of the intended end user is only the first step in the health care performance improvement cycle. Developers and organizational leadership must also employ implementation strategies to promote uptake and use of the dashboard, such as the identification and involvement of “champions,” ongoing training of end users, and changes in policy that mandate or incentivize dashboard use [20]. As development and maintenance of data-rich business intelligence tools, like dashboards, can be time- and resource-intensive, it is essential that these tools both function effectively and result in measurable improvements. Iterative evaluation of dashboard performance throughout development and implementation and beyond are critical to identify user- and system-level barriers to use as well as potential errors that may only be identified after extended use.

In this scoping review, we will survey peer-reviewed literature to describe the contexts in which dashboards have been used in health care settings, as well as how they were developed, implemented, and evaluated.

### Aims and Comparison With Prior Work

This scoping review will provide a narrative overview of design elements and characteristics of health care dashboards, including where they exist geographically, the intended end users, information presented, whether/how the end user and setting impact dashboard design, and the processes used for development, implementation, and evaluation. Although previous reviews on health care dashboards have focused on identifying important design features and effectiveness of dashboards in improving patient outcomes and clinician satisfaction [11,14,15,21,22], an updated review of *how* dashboard tools are used, and *by whom* will provide meaningful insight into how intended end user and setting impact the design, development and implementation of the dashboard (ie, the relationship of form and function). This information is essential to provide insights into (1) *how* and *why* dashboards work in different settings for different users, to allow relevant stakeholders to make more informed decisions about where to implement, and (2) how to effectively design dashboards based on their intended purpose and target audience. Given the rapidly evolving field of health informatics, the scoping review will also provide insight into the latest trends in dashboards, from initial conception and development through implementation and evaluation. Previous reviews of dashboards have included articles published only as recently as 2017 [11,14,21-23].

## Methods

### Study Design

The aims of this study can be best accomplished through a scoping review, which differs from a systematic review in that scoping reviews generally have a broader scope and are exploratory, not requiring critical quantitative appraisal of synthesized findings [24,25]. For this study, we will follow the framework for conducting scoping reviews developed by Arksey & O’Malley [26] and further refined by Levac et al [27]. A description of each step is provided below.

### Step 1. Identifying the Research Questions

The key research questions, which were established through a process of team discussions and preliminary searches of the literature on health care dashboards, are as follows:

1. What design features are most frequently incorporated in health care dashboards?
2. For what purposes are dashboards developed in health care settings?
3. Where, and by whom, are dashboards used?
4. What processes and/or frameworks are used for development, implementation, and evaluation of dashboards?

### Step 2. Identifying Relevant Studies

We searched MEDLINE, Embase, Web of Science, and the Cochrane Library databases in July 2020 for relevant articles using comprehensive search strategies for each database that were developed in collaboration with a research librarian (MLC) and are available in Multimedia Appendix 1. These databases were selected since they represent a broad sample of literature relevant to the health sciences. Search terms included a variety of keywords and medical subject headings (MeSH) related to clinical health care and information technology. Search strategies were developed around the following key terms: “dashboard,” “information technology,” “healthcare,” “electronic health record,” “electronic medical record,” “quality,” “safety,” “key performance indicators,” “decision making,” “decision support,” “benchmark,” and “informatics.” Boolean operators “AND,” “OR,” and “NOT” were used to construct each search, with “NOT” operators used to reduce the number of results related to automotive and learning analytics dashboards. No date, language, or other restrictions were imposed in the database searches. Grey literature sources were not searched.

### Step 3. Study Selection

All articles retrieved by the search were imported into and initially reviewed using Covidence [28], a screening and data extraction tool adopted by Cochrane in 2015 as the standard platform for producing Cochrane Reviews. In addition, 2 of 4 authors (DH, ADR, MLC, OJG) independently screened all titles and abstracts to identify potentially eligible studies. All articles that mentioned use of a “dashboard” in a health care setting were reviewed in full text and are currently undergoing duplicate review by 2 of 7 authors (DH, ADR, MLC, OJG, ANK, RG, AR) to determine eligibility, applying the inclusion and exclusion criteria listed in Textbox 1. We excluded articles published prior to 2015 and those describing dashboards that were not successfully used in routine workflow or were only used in a pretesting environment. We believe these exclusions are justified as rapid advancements in technology warrant a focus on newer research that is more likely to be reproducible. Additionally, limiting our analysis to dashboards that were successfully implemented or used outside of a pretesting environment provides a clearer view of existing barriers and facilitators to designing and implementing dashboards in real-world practice. Any disagreements that arise during full-text screening will be resolved through adjudication by a third author. For any studies that are reviewed in full text but not deemed eligible for inclusion in the scoping review, a reason for exclusion will be documented and provided with the results of the scoping review in a PRISMA (Preferred Reporting Items for Systematic Reviews and Meta-Analyses) flow diagram.

**Eligibility criteria for full-text review.**

**Inclusion criteria**

- Peer-reviewed articles that describe the development, implementation, and/or evaluation of a dashboard used in a health care setting outside of a pretesting environment. Health care settings include clinics, hospitals, health systems, or any other settings where medical care is provided. Both quantitative and qualitative evaluations of dashboards will be included.

**Exclusion criteria**

- Non–English language publication
- Articles published prior to 2015
- Articles that describe pretesting of pilot or prototype dashboards that were not successfully implemented or used outside of a testing environment
- Articles that describe public health dashboards used for geographic tracking of disease or comparing city- or country-level data not used for clinical or management-level decision-making in a health care setting where patient care is provided
- Articles that describe dashboards used in undergraduate medical education, or in educational contexts where there is no direct association with patients, patient care, or facility management
- Articles for which the full-text manuscript is unavailable

### Step 4. Charting the Data

A preliminary list of data elements for charting is presented in Textbox 2. However, in accordance with recommendations from Levac et al [27], an iterative process will be used to identify additional elements for data extraction and analysis as the study progresses. Using an iterative process improves the quality of the review by allowing reviewers to gain familiarity with included studies and add or revise data extraction elements accordingly. A standardized data extraction form will be developed and reviewed by all authors. The form will be pilot tested by two authors who will independently complete data extraction for a subset of articles to ensure consistency among extractors. Once a high level of agreement is achieved between extractors, the pilot extraction form will be approved, and two authors will independently extract data from each included study. Any disagreements in data extraction will be resolved by discussion between the two authors; if the reviewers are unable to reach consensus, a third author will serve as arbiter.

**Preliminary list of data extraction elements.**

**Article information**

- Title
- Author
- Publication year
- Journal
- Study type

**Contextual factors**

- Geographic location of the described dashboard
- Health care setting
- Intended end user(s)

**Primary purpose or goal of the dashboard**

- Reason stated for development or use of dashboard

**Development**

- Software used
- Framework(s) used to guide development or pretesting
- Usability testing conducted
- Involvement of users in development process

**Implementation**

- Adjunct strategies used in conjunction with dashboard (such as academic detailing, audit and feedback, or financial incentivization)
- Identification of potential barriers and facilitators to use of dashboard prior to implementation
- Identification and involvement of champions
- Training of stakeholders or distribution of educational materials on how to use the dashboard
- Protocol or policy changes that mandate use of the dashboard

**Evaluation**

- Type of evaluation (qualitative or quantitative)

**Design features**

- Format (including delivery channel and timing)
  Frequency of data updates
  Use of visual elements
  Delivery channel (eg, website, email, wall display)
- Information content
  Descriptions of performance summary data (including indicators, time intervals, comparators, and their performance levels)
  Patient lists (typically patients who have actionable data, such as guideline-discordant care; “yes” or “no”)
  Patient-level data (“yes” or “no”)
  Recommended actions (“yes” or “no”)
  Metrics or evaluation based on benchmarks established by accrediting bodies, health care payer organizations, or national guidelines (“yes” or “no”)
- Functionality (“yes” or “no”)
  Multilevel presentation of data
  Interface customizability
  Goal setting/action planning
  Task performance (ie, ordering, flagging, prescribing)

### Step 5. Collating, Summarizing, and Reporting the Results

Data extraction will be performed using Microsoft Excel (Microsoft Corp). The data elements for each dashboard identified will be displayed and coded in a spreadsheet, which will be used for analysis, mainly counts. This scoping review will follow the PRISMA-ScR (Preferred Reporting Items for Systematic Reviews and Meta-Analyses extension for Scoping Reviews) checklist [29] for reporting of methods and outcomes.

## Results

In July 2020, electronic database searches were completed using the search strategies outlined in Multimedia Appendix 1, and 5188 results were retrieved and imported into Zotero reference management software (version 5.0.96.2; Corporation for Digital Scholarship) for management of records and retrieval of full-text articles prior to upload into Covidence online screening software [28]. After removal of duplicate results in Covidence [28], there were 3306 articles identified for title and abstract screening. A total of 2019 articles were retrieved for full-text review and will be reviewed in duplicate for eligibility. We aim to finish the review and draft the final report by mid-2022. Findings will be summarized in a narrative fashion while employing use of tables and graphs to illustrate key characteristics of dashboards in health care and will be submitted for publication along with the completed PRISMA-ScR reporting checklist.

## Discussion

### Future Planned Work

Currently, available literature lacks standard, consensus hierarchical descriptions of the different types of health care dashboards in use and their distinct design and implementation processes [15,30]. As the use of dashboards continues to increase, it is important for stakeholders to be able to communicate effectively with the designers and users of these tools. The authors intend to use the findings of this scoping review to inform the development of a taxonomy of the various types of dashboards a health care organization may choose to employ. This taxonomy will identify the relevant design elements that each type of dashboard includes to inform evidence about health care dashboard usability and purpose of use, and stakeholders, including end users. Finally, the review will provide evidence of the extent to which rigorous practices are used in the development, evaluation, and implementation of health care dashboards, each of which ultimately contributes to a dashboard’s success.

The findings of this scoping review will additionally inform the design of a future meta-analysis and meta-synthesis of dashboard evaluations, if possible, in consideration of the heterogeneity of the studies identified in this scoping review.

### Limitations

This scoping review methodology has several limitations. First, the search strategy does not include grey literature or conference abstracts since these are expected to provide insufficient detail for the data elements we plan to extract. This may cause some dashboards described in government and committee reports, dissertations, and conference proceedings to be overlooked. However, since the data extracted will mainly be summarized, and since we are not evaluating any causal effects or performing quantitative analyses, which would be more susceptible to publication bias, this will not be a major limitation. Second, because of the inclusion criteria, our findings will be most applicable to dashboards used in settings that provide direct health care; they will be less informative about public health tracking dashboards, including those used to monitor the COVID-19 pandemic and to perform contact tracing [31,32].

### Conclusion

Health information technology continues to rapidly change the way health care organizations operate, and dashboards are an increasingly common tool. It is essential that key stakeholders have a clear understanding of what dashboards are, and which features are essential to specific end users for dashboard development. This scoping review will advance the field of health informatics by providing organizational leaders, clinical staff, dashboard developers, and quality improvement researchers with a clear and concise overview of the literature in this field, and by highlighting research gaps.

## Appendix

Multimedia Appendix 1 Database search strategies.

## Abbreviations

MeSH: medical subject headings
PRISMA: Preferred Reporting Items for Systematic Reviews and Meta-Analyses
PRISMA-ScR: Preferred Reporting Items for Systematic Reviews and Meta-Analyses extension for Scoping Reviews
