# Supplementary material for: Dashboards in Health Care Settings: Protocol for a Scoping Review
Source: JMIR Res Protoc. 2022 Mar 2;11(3):e34894. doi: 10.2196/34894 (PMC8928055; doi:10.2196/34894)
Supplement: Multimedia Appendix 1 [file resprot_v11i3e34894_app1.docx]

**Appendix 1. Database Search Strategies**

| **Database** | **Search Strategy** |
| --- | --- |
| **Medline – OVID** | ((informatics or "information technology" or "health IT" or "electronic health record$" or "electronic medical record$" or "hospital management system$" or "clinical management system$" or inpatient$ or hospital$ or outpatient$ or ambulatory or emergency or "service improvement" or benchmark or "decision support" or "decision making" or performance or productivity or outcome$ or "key performance indicator$" or KPI or workflow or clinical$ or clinic$ or "health care" or safety or quality or "point of care" or alert$ or reminder$ or monitor$) and dashboard$).ab,ti  OR  (exp medical informatics/ OR exp nursing informatics/ OR exp medical records systems, computerized/ OR exp "health care quality access and evaluation"/) AND dashboard$.ab,ti  OR  (dashboard$ not (car or cars or automobile$ or vehicle$ or traffic or police or "learning analytic$")).ab,ti. |
| **EMBASE** | (informatics:ab,ti OR 'information technology':ab,ti OR 'health it':ab,ti OR "electronic health record$":ab,ti OR "electronic medical record$":ab,ti OR "hospital management system$":ab,ti OR "clinical management system$":ab,ti OR inpatient$:ab,ti OR hospital*:ab,ti OR outpatient$:ab,ti OR ambulatory:ab,ti OR emergency:ab,ti OR 'service improvement':ab,ti OR benchmark:ab,ti OR 'decision support':ab,ti OR 'decision making':ab,ti OR performance:ab,ti OR productivity:ab,ti OR outcome$:ab,ti OR "key performance indicator$":ab,ti OR kpi:ab,ti OR workflow$:ab,ti OR clinic*:ab,ti OR 'health care':ab,ti OR safety:ab,ti OR quality:ab,ti OR 'point of care':ab,ti OR alert$:ab,ti OR reminder$:ab,ti OR monitor*:ab,ti) AND dashboard$:ab,ti  OR  ('health care quality'/exp OR 'information processing'/exp) AND dashboard$:ab,ti  OR  dashboard$:ab,ti NOT (car:ab,ti OR cars:ab,ti OR automobile$:ab,ti OR vehicle$:ab,ti OR traffic:ab,ti OR police:ab,ti OR "learning analytic$":ab,ti) |
| **Web of Science** | TS=((informatics or "information technology" or "health IT" or "electronic health record$" or "electronic medical record$" or "hospital management system$" or "clinical management system$" or inpatient$ or hospital$ or outpatient$ or ambulatory or emergency or "service improvement" or benchmark or "decision support" or "decision making" or performance or productivity or outcome$ or "key performance indicator$" or KPI or workflow or clinical$ or clinic$ or "health care" or safety or quality or "point of care" or alert$ or reminder$ or monitor$) and dashboard$)  OR  TS=(("health care quality" OR "information processing") AND dashboard$)  OR  TS=  (dashboard$ not (car or cars or automobile$ or vehicle$ or traffic or police or "learning analytic$")) |
| **Cochrane Library** | ((dashboard OR dashboards)):ti,ab,kw |
